# Dental Physiology

**Published:** 1873-07

**Authors:** Henry. S. Chase


### ARTICLE V.

### *Dental Physiology.*

BY HENRY S. CHASE, ST. LOUIS.

That dentition is a natural physiological process ought not to be doubted. Many, indeed I think the majority of writers on medicine, have classed it among the *causes* of infantile diseases. That there are very many diseases peculiar to the period of dentition cannot be denied. But they are only so, owing to the remarkably impressible condition of the infantile vitality or constitution, just as a young and succulent vegetable growth is easier marred or injured than that which is more matured. And as the young vegetable growth more quickly recovers from mechanical injury than the older one, so do the constitutions of children more quickly recuperate after the morbid cause is removed than do those of adults.

It is not strange that these facts are so patent, when we consider the wonderful activity of the processes of Nutrition, growth and disintegration. In a normal condition the frequency of the heart's action indicating the rapidity of the sanguineous circulation, is a true index of the rapid changes taking place in the young growing tissues.

When from a sufficient cause a cell is changed from its natural physiological condition, whatever that may be, its proliferation, both as to rapidity and extent, is in proportion to the quickness of the chemico-vital changes of the individual whether he be young or old. And thus it is that the new being in its flushness of chemico-vital processes coming in contact with moibific agents inimical to health and life of older and more stable organizations, is itself easily impressed with their forms, and multiplies them with the force natural to its vitality.

On the other hand we stoutly contend that no natural physiological process in one set of organs should or does necessarily induce disease in other portions of the body which is in a natural condition.

The laws of nature are all in mutual relation or harmony, and it would be a reflection on divine wisdom to admit any proposition to the contrary. Therefore, I reiterate the fact that dentition is not necessarily a cause of disease. But the converse is true; namely, that disease is a fruitful source of mal-dentition. What is usually termed dentition is the process of Eruption or passage of the teeth through the gums.

But I think a better definition would be this, namely, *the formation, growth and eruption of the teeth.* And on this basis I make the following remarks:

The derangements of dentition commence *early* in GESTATIVE UTERINE LIFE. We may not place the period later than the fourth month, or when the dentification or calcification of the germs is fairly under way.

The foundation of mal-dentition is often laid still earlier than this, namely, at the moment when the OVUM is impressed with the vitality of the spermatozoa, and the NEW BEING is at once created, and receives its law of development at that same instant, with all its peculiarities stamped upon its yet unshapen members, subject only to modifying causes affecting nutrition. For on the physiological nutrition of the new being depends the strength, permanence, and vital forces of its individual organs.

MAL-NUTRITION depends upon

1st. The non-ingestion of proper food.

2d. The non-ingestion of sufficient food.

3d. Non-digestion or imperfect digestion.

4th. Non-assimilation, or imperfect assimilation.

The two latter are often produced by Idiopathic, or constitutional disease in the mother.

Tissues cannot be formed without material. The *mother* must provide this during the whole period of gestation and lactation, not only for her own body, but for that of her growing child. In health, nature furnishes the mother with an *extra* appetite for food, causing her to eat an extra diurnal amount, corresponding to the demands of the new being. If food of natural proportions, or that which has not been robbed of its *natural elements* is used by the mother, the desirable end will be accomplished. But if this is *not* the case; if, for instance, the *starch* of wheat is used as a factor of nutrition to the exclusion of the gluten and the inorganic elements which the whole berry contains, then all the tissues of the body must suffer.

Transformations of tissue must go on whether food is ingested or not; and disintegration of every tissue of every organ will daily take place in very regular and definite proportions both in conditions of nutrition and starvation.

Whatever chemical analysis proves to be the constituents of any tissue, must be furnished by the blood, or that tissue will diminish in weight, whether it be muscular, cartilaginous, osseous or other.

Starch cannot take the place of gluten, neither will soda or potassa interchange with lime.

In my Report on Dental Physiology, to the American Dental Association for the session of 1865, I endeavored to show that the fine flour of wheat is unfit for the principal food of an expectant or nursing mother, as indeed it is for any other person. I think I succeeded in proving that it is very deficient in those inorganic elements which the bones and teeth particularly demand for their formation and preservation. I also endeavored to show that the universal use of this article of diet in the United States is the *principal* cause of defective teeth in this country.

There is no *discord* in the laws of nature; for every physiological necessity there are means for its satisfaction. The Creator has placed in every variety of food the proximate elements required for the nutrition of the body. The ingenuity of man alone has sacrificed the good and the natural to the gratification of his depraved tastes. The whole brute creation obeys the laws of nature, and reaps the rewards of health.

The practical lesson which I wish to impress on the mind is this, namely: that those who intend to be parents should have their own dentures in a sound and healthy condition *before* they cause the conception of a new being. As peculiar conditions of the system are transmitted from one generation to another, parents cannot be too careful that no taint of theirs shall curse their offspring. Acting in accordance with the laws of enlightened hygiene will ensure this.

In addition to this the *constitutional* diseases which impress their types on progeny with such fearful constancy must be cured. The most dreadful of all these is syphilis. There is none more fatal to dental nutrition and growth than this. Its unrelenting grasp holds dental development in check to the third and fourth generation. It may almost daily be seen in notched incisors, eroded enamel and defective dentine.

Mercurial disease is hardly less fatal to the teeth and causes similar pathological sequences.

Consumption, as well as the two diseases named, produces dwarfed teeth. Consumption causes pearly looking teeth, with calcification complete. These decay readily, and cut easily with an excavator even when not decayed.

If the teeth of parents are in a healthy condition, even if many of them have been decayed and are now plugged, and the general health is good, and no constitutional taint is present in the body, there need be no fear of transmitting to the offspring the influences of imperfectly organized teeth, provided the normal acts of nutrition are not afterward thwarted by disregarding the laws of dental hygiene. To this end the mother must ingest sufficient food containing osteogenous and dental constituents in *nature's proportions* in order to furnish the child which she carries, with all its needs.

There is no mystery or difficulty about this. Excepting in substituting fine flour for that which is *unbolted* there can hardly be a mistake. Lean meats, meal of all the cereals, the legumens, garden roots, and fruits are all suitable. And then there is milk, which deserves letters of gold, cheese and butter. Fermented and spirituous liquors can add nothing unless they idiosyncronise and promote digestion and absorption. Cleanliness of the skin; purity of air; sunlight, together with exercise in the open air, become almost indispensable adjuncts to diet in dental or general hygiene in civilized life.

If the type of American teeth is ever to be improved, so that they may become a *blessing* to the majority instead of as now a curse, dental physiology must point to conception and gestation as the periods for the commencement of those important measures which are to accomplish the great work.

Here is not only legitimate work, but the *real* work to do. If "an ounce of prevention is worth more than a pound of cure," then here is the *remedy* which faithfully applied will prove a blessing to this nation, and perhaps to the world.

As gestation is as much a physiological process as sleeping or cutting teeth, there are no diseases necessarily consequent upon it. There are few ailments common to gestation, of which I will briefly speak. The first is

MORNING SICKNESS.

This is certainly not a disease, but is just as truly regarded by the mother as an ailment, at least, from which she feels that she would only be too happy to be relieved. Yet it probably is not usually cut short or cured by *medicine* without detriment to the mother, for it is undoubtedly a physiological act; a preparatory act for the better nutrition of the system. Is it not an effort of nature to prepare the body for a more perfect assimilation of food, to increase the appetite, to strengthen the powers of digestion, thereby furnishing the embryo with abundance of material for development and growth? Women in savage life are subject to this condition, so are some animals; at least I have observed it in cats and dogs. Therefore, unless this condition becomes abnormally developed, by latent constitutional psora, or bad habits, it should not be interfered with.

Constipation is another ailment common to gestation among flour eaters. Those who use unbolted wheat meal rarely suffer in this respect.

Hemorrhoids or piles is another trouble common to the same class of persons. Although in advanced stages of gestation there is a natural tendency to produce prolapsus of the large intestine, yet it does not take place where the principles of enlightened hygiene are obeyed. Piles is not an idiopathic disease, but only a symptom consequent on a disturbance in a more distant portion of the body.

A consideration of these ailments in all their bearings would carry me too far from the main subject, which is not therapeutical, but hygienic.

It is very important to secure for the expectant mother uninterrupted good health during the whole period of gestation and lactation; therefore ailments or diseases must be promptly met by all the hygienic and therapeutical means at our command.

Whenever sickness is serious enough to affect ingestion and digestion even for the space of one month it will inevitably leave its marks upon the microscopical structure of the teeth. The proximate cause is innutrition. When this takes place early in gestation, say before the fifth month, the dental germs which are in a soft and pulpy state are arrested in growth and receive an impression which often dwarfs them in size. Not only does this happen to the milk teeth, but the bulbs of the permanent ones are affected also. But the latter undoubtedly are more seriously affected after birth up to the fifth year by the causes mentioned.

It has often been observed that some children are "small of their age" up to five or six years or older, and that they afterward "take a start" and grow to the full size of womanhood or manhood which their parentage warrants. Now as teeth cannot enlarge in diameter after the calcification of the enamel and the coronal ends of the dentine tubes, they necessarily remain of the diminutive size which they are forced to take by starvation. And thus it is that we so often observe in the mouths of adults teeth which properly correspond in size to those of the milk teeth. Especially is this to be observed in lateral incisors, canines and bicuspids. I would suggest that the contour lines or shadings so often seen in the dentine of these teeth are the result of arrested development, showing the size and shape of the bulbs at the time. Sometimes there are several of these in one tooth, corresponding to successive arrests of growth.

The lacunæ or interglobular spaces in dentine are very likely the result of arrested development in the dentinal corpuscles, from a lack of nutrition also.

That these effects may be afterward remedied by good health and a better nutritive supply I do not doubt. But the importance of the mother's health for the perfect development and growth of the dental tissues is hereby shown.

Dental calcification is a long process, and continues from the fourth month of intra-uterine existence far beyond adult life. It is seen not only in the increasing hardness of the teeth as age progresses, but also in the actual transformation of the dental pulp into a substance resembling bone. A *calcification* in fact of its connective tissue.

At the period when the new being is ushered into a new world no teeth are yet erupted, for none are fully formed, and therefore nature kindly furnishes the child with a pabulum for its nourishment, requiring neither mastication nor effort of the will to fit it for its digestion.

Notwithstanding the changed relations of mother and child as regards position and mode of nourishment, yet the fact still remains as plain as before that her blood should be a magazine plentifully supplied with lime salts for the continued development and perfection of the dental organs. From birth until weaning is the period in which the *drain on the mother's blood* is most severe, and as lactation goes on, the teeth become rapidly diseased in many cases, and caries of the dentine is more or less developed according to the present and previous hygienic habits, and health of the mother. The activity of dental decay in these unfavorable cases is in strong contrast with that of unmarried women and men. Fractures of the bones also heal with difficulty owing to the diminished quantity of lime phosphate in the blood. Not only do the osseous and dental tissues of the mother suffer, but the whole system is under a general *malaise* consequent on the lack of these salts in *every* tissue of the body. The brain, which uses up so much phosphoric acid in the operations of the mind, is dull and feeble, and the muscles pale and flabby, and incapable of their normal work.

At this period it is customary to apply to the dentist for professional aid, only however to solicit his *surgical* and *mechanical* skill ; *rarely* to ask advice to hygiene. Who is to blame? Without doubt the dental profession which has not yet come up to the standard of physiological science which the age demands.

When we have all learned the necessities of a mother famishing for the lack of the necessary elements of nutrition, mothers themselves will become aware of their needs through our instructions at the dental chair, the social circle and the public press, and will apply to those for aid whose duty it is to render it.

A mother in this condition should not be sent away with patched teeth merely, but with that knowledge which shall gradually build up again her disintegrated tissues as rapidly as physiological laws will admit. This is not impracticable, it has often been done.

*To be Continued.*

